# Acute hypopituitarism associated with periorbital swelling and cardiac dysfunction in a patient with pituitary tumor apoplexy: a case report

**DOI:** 10.1186/s13256-017-1371-7

**Published:** 2017-08-24

**Authors:** Nobumasa Ohara, Yuichiro Yoneoka, Yasuhiro Seki, Katsuhiko Akiyama, Masataka Arita, Kazumasa Ohashi, Kazuo Suzuki, Toshinori Takada

**Affiliations:** 1 Department of Endocrinology and Metabolism, Uonuma Institute of Community Medicine, Niigata University Medical and Dental Hospital, 4132 Urasa, Minamiuonuma, Niigata 949-7302 Japan; 2 Department of Neurosurgery, Uonuma Institute of Community Medicine, Niigata University Medical and Dental Hospital, Niigata, Japan; 3 Department of Cardioavascular Medicine, Uonuma Institute of Community Medicine, Niigata University Medical and Dental Hospital, Niigata, Japan; 4 Department of Respiratory Medicine, Uonuma Institute of Community Medicine, Niigata University Medical and Dental Hospital, Niigata, Japan

**Keywords:** Hypopituitarism, Pituitary tumor apoplexy, Central hypothyroidism, Adrenal insufficiency, Hypertension, Levothyroxine, Hydrocortisone

## Abstract

**Background:**

Pituitary tumor apoplexy is a rare clinical syndrome caused by acute hemorrhage or infarction in a preexisting pituitary adenoma. It typically manifests as an acute episode of headache, visual disturbance, mental status changes, cranial nerve palsy, and endocrine pituitary dysfunction. However, not all patients present with classical symptoms, so it is pertinent to appreciate the clinical spectrum of pituitary tumor apoplexy presentation. We report an unusual case of a patient with pituitary tumor apoplexy who presented with periorbital edema associated with hypopituitarism.

**Case presentation:**

An 83-year-old Japanese man developed acute anterior hypopituitarism; he showed anorexia, fatigue, lethargy, severe bilateral periorbital edema, and mild cardiac dysfunction in the absence of headache, visual disturbance, altered mental status, and cranial nerve palsy. Magnetic resonance imaging showed a 2.5-cm pituitary tumor containing a mixed pattern of solid and liquid components indicating pituitary tumor apoplexy due to hemorrhage in a preexisting pituitary adenoma. Replacement therapy with oral hydrocortisone and levothyroxine relieved his symptoms of central adrenal insufficiency, central hypothyroidism, periorbital edema, and cardiac dysfunction.

**Conclusions:**

Common causes of periorbital edema include infections, inflammation, trauma, allergy, kidney or cardiac dysfunction, and endocrine disorders such as primary hypothyroidism. In the present case, the patient’s acute central hypothyroidism was probably involved in the development of both periorbital edema and cardiac dysfunction. The present case highlights the need for physicians to consider periorbital edema as an unusual predominant manifestation of pituitary tumor apoplexy.

## Background

Pituitary tumor apoplexy (PTA) is a rare clinical syndrome caused by acute, often spontaneous, hemorrhage or infarction in a preexisting pituitary adenoma [1, 2]. The rapid enlargement of the pituitary tumor compresses the adjacent parasellar structures and typically manifests as an acute episode of headache, altered mental status, visual disturbance, cranial nerve palsy, and endocrine pituitary dysfunction. PTA can be life-threatening, and it requires prompt diagnosis and treatment. Because not all patients present with classic symptoms, it is pertinent to appreciate the clinical spectrum along which PTA can present.

Edema surrounding the eyes, called periorbital edema, can be caused by infections, inflammation, trauma, allergy, and kidney or cardiac dysfunction. It can also result from endocrine disorders such as hypothyroidism, and there are reported cases of bilateral periorbital edema associated with acute primary hypothyroidism [3–5]. However, few studies have investigated patients with hypopituitarism who presented with periorbital edema.

Here, we report the case of an elderly patient with PTA who presented with severe bilateral periorbital edema associated with acute anterior hypopituitarism in the absence of typical PTA symptoms.

## Case presentation

An 83-year-old Japanese man was admitted to our hospital in July 2016 because of anorexia, loss of bodyweight, fatigue, and lethargy. He had a family history of paternal hypertensive cerebral hemorrhage. The patient had a history of right lung adenocarcinoma that was surgically removed in his seventies. He also had allergy to contrast medium. The patient was diagnosed with essential hypertension at 73 years of age, and started antihypertensive medication (5 mg/day oral amlodipine) at a local clinic. In the spring of 2016, he was 169 cm tall, weighed 72 kg, and had an office blood pressure (BP) of 135/70 mmHg under antihypertensive treatment. Two months before admission, the patient developed acute anorexia, fatigue, and lethargy, and had difficulty opening his eyes because of marked swelling of the bilateral upper and lower eyelids, so he visited the clinic. He weighed 66 kg, had a BP of 100/50 mmHg, and presented with marked periorbital edema accompanied by facial swelling without itching, pain, redness, erythema, or warmth; there were no skin lesions at other sites and no peripheral edema. The antihypertensive mediation was discontinued because of his low BP, and he started diuretics (20 mg/day oral furosemide and 25 mg/day spironolactone) for his periorbital edema. Within a week, he experienced a partial improvement in his periorbital edema and facial swelling. Because blood chemistry showed low serum free thyroxine (FT_4_; 0.49 ng/dL) and low-normal serum thyroid-stimulating hormone (TSH; 0.73 μIU/mL) levels, the patient started thyroid hormone replacement therapy with oral levothyroxine at a dose of 50 μg/day, which was subsequently titrated up to 100 μg/day, and the diuretics were discontinued. The patient experienced a complete resolution of his periorbital edema and facial swelling within 3 weeks, with normalization of serum FT_4_ levels (0.96 ng/dL). However, his anorexia, fatigue, and lethargy worsened. Because his serum cortisol (0.5 μg/dL) and plasma adrenocorticotropic hormone levels (ACTH; 4.3 pg/mL) measured mid-morning were low, the patient was referred to our hospital for further endocrine examinations.

On physical examination at admission, our patient was alert, and his bodyweight, body temperature, BP, pulse rate, and oxygen saturation by pulse oximeter (SpO_2_) on room air were 61.5 kg, 36.4 °C, 93/40 mmHg, 81 beats per minute, and 100%, respectively. His pupil diameter, light reflex, eye movement, visual field, and facial sensations were all normal. There was no skin pigmentation, proptosis, exophthalmos, periorbital edema, gynecomastia, chest rales, abdominal tenderness, muscle pain, or peripheral edema. A grade II/VI apical systolic regurgitant murmur was detected. Laboratory findings (Table 1) showed mild anemia, low serum cortisol (1.8 μg/dL) levels, and high serum C-reactive protein (5.53 mg/dL), prolactin (28.5 ng/mL), and plasma brain natriuretic peptide (BNP; 421.6 pg/mL) levels. His serum TSH level (0.03 μIU/mL) was low, but serum FT_4_ (0.98 ng/dL) and free triiodothyronine (FT_3_; 3.73 pg/mL) levels were normal under thyroid hormone replacement therapy with levothyroxine (100 μg/day). Tests for antithyroglobulin antibodies, thyroid peroxidase antibodies, second-generation TSH-binding inhibitor immunoglobulins, and antinuclear antibodies were negative. An ultrasound detected no abnormalities in the thyroid gland. Additionally, chest and abdominal computed tomography (CT) scans showed no abnormalities in the lung, liver, kidney, and adrenal glands, but mild cardiomegaly and bilateral pleural effusion were observed. A 12-lead electrocardiogram showed occasional premature ventricular contractions with no abnormal waveform. An echocardiogram showed a hypokinetic area in the anterior wall of the left ventricle with a left ventricular ejection fraction (LVEF) of 55%, moderate mitral valve regurgitation, and mild pericardial effusion.

**Table 1:** Laboratory findings on admission (July 2016)

Hematology		
Red blood cells	363 × 10^4^/μL	(435–555)
Hemoglobin	10.5 g/dL	(13.7–16.8)
Hematocrit	32.3%	(40.7–50.1)
White blood cells	4100/μL	(3300–8600)
Platelets	18.7 × 10^4^/μL	(15.8–34.8)
Blood chemistry		
Total protein	6.5 g/dL	(6.6–8.1)
Albumin	3.7 g/dL	(4.1–5.1)
Aspartate aminotransferase	19 IU/L	(13–33)
Alanine aminotransferase	15 IU/L	(10–42)
Creatine kinase	57 IU/L	(45–163)
Urea nitrogen	16.3 mg/dL	(8.0–18.4)
Creatinine	1.01 mg/dL	(0.65–1.07)
Sodium	138 mmol/L	(138–145)
Potassium	3.9 mmol/L	(3.6–4.8)
Chloride	103 mmol/L	(101–108)
C-reactive protein	5.53 mg/dL	(<0.14)
Prothrombin time	105.8 seconds	(70–130)
APTT	27.9 seconds	(24–32)
Fibrinogen	278.1 mg/dL	(200–400)
Casual plasma glucose	106 mg/dL	(70–139)
Glycated hemoglobin	4.9%	(4.6–6.2)
Brain natriuretic peptide	421.6 pg/mL	(<18.4)
Plasma osmolality	281 mOsm/L	(275–290)
Plasma arginine vasopressin	2.3 pg/mL	
Prolactin	28.5 ng/mL	(3.6–12.8)
Thyroid-stimulating hormone	0.03 μIU/mL	(0.50–5.00)
Free triiodothyronine	3.73 pg/mL	(2.30–4.00)
Free thyroxine	0.98 ng/dL	(0.90–1.70)
Adrenocorticotropic hormone	16.3 pg/mL	(7.2–63.3)
Cortisol	1.8 μg/dL	(4.5–21.1)
Dehydroepiandrosterone sulfate	138 ng/mL	(50– 2,530)
Aldosterone	6.2 ng/dL	(3.0–15.9)
Plasma renin activity	0.2 ng/mL/h	(0.2–2.3)
Noradrenaline	0.70 ng/mL	(0.10–0.50)
Adrenaline	0.06 ng/mL	(0–0.10)
Dopamine	0.01 ng/mL	(0–0.03)
Urinalysis		
Specific gravity	1.024	(1.005–1.020)
pH	5.5	(5.5–7.5)
Glucose	Negative	
Protein	Negative	
Occult blood	Negative	
Inflammatory cells	Negative	

The reference range for each parameter is shown in parentheses

Blood samples were taken with the patient in the supine position at 9 AM of the day of admission. The patient underwent thyroid hormone replacement therapy with oral levothyroxine (100 μg/day)

*APTT* activated partial thromboplastin time

**Fig. 1:**
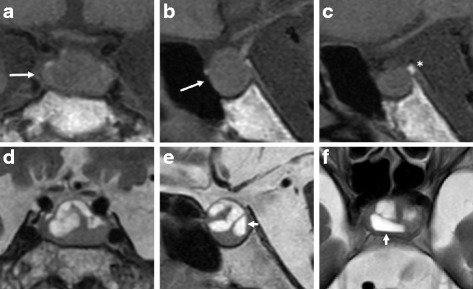
Magnetic resonance imaging of the brain (July 2016). Plane T1-weighted images (**a**: coronal plain, **b**: sagittal plain) showing a 2.5-cm pituitary tumor (*arrows*) and deformed hypophyseal stalk, with no compression on the optic chiasm. The physiological high-intensity signal (*) was found in the posterior pituitary gland (**c**: sagittal plain). T2-weighted images (**d**: coronal plain, **e**: sagittal plain, **f**: transverse plain) revealed a mixed pattern of solid and liquid components in the pituitary tumor, with fluid-fluid levels (*short arrows*)

Our patient was suspected to have both central hypothyroidism and adrenal insufficiency (AI). Because thyroid hormone replacement alone could exaggerate AI under such a condition [6], the oral levothyroxine was discontinued on the day of admission. A rapid ACTH stimulation test (Table 2) showed incomplete cortisol secretion in the presence of an adequate aldosterone response. A combined anterior pituitary stimulation test (Table 3) showed a decreased response of growth hormone (GH) to growth hormone-releasing factor (GRF) and decreased response of follicle-stimulating hormone (FSH) and luteinizing hormone (LH) to luteinizing hormone-releasing hormone (LHRH). An apparently adequate response of ACTH was observed following corticotropin-releasing hormone (CRH) administration, but the cortisol response was reduced. Growth hormone-releasing peptide 2 administration (Table 4) yielded a decreased GH release but an apparently adequate release of ACTH; however, the cortisol response was insufficient. A prolonged ACTH stimulation test (Table 5) showed adequate cortisol secretion. Magnetic resonance imaging (MRI) of the brain (Fig. 1) revealed a 2.5-cm pituitary tumor with the hypophyseal stalk deformed. The tumor contained a mixed pattern of solid and liquid components with fluid-fluid images on T2-weighted images consistent with the subacute phase of an intratumoral hemorrhage. Brain magnetic resonance angiography detected no abnormalities. These findings indicated a diagnosis of anterior hypopituitarism with PTA due to hemorrhage in a preexisting pituitary adenoma [6–9]. As the patient had no PTA symptoms, such as headache, altered consciousness, visual impairment, or cranial nerve palsy, pituitary surgery was not indicated. Our patient was scheduled to receive medical management with hormone replacement therapy; he started corticosterone replacement therapy with oral hydrocortisone (20 mg/day) for his central AI on day 11 of admission. A thyrotropin-releasing hormone (TRH) stimulation test (Table 6) performed on day 21 of admission reveled low release of TSH under conditions of low serum FT_3_ and FT_4_ levels, confirming the diagnosis of central hypothyroidism. Our patient resumed replacement therapy with oral levothyroxine (75 μg/day) for his central hypothyroidism on day 22 of admission. He regained his appetite and vitality, and was discharged on day 25 after admission.

**Table 2:** Endocrinological investigation: Rapid adrenocorticotropic hormone stimulation test in July 2016 (day 3 after admission)

	Reference range for basal value	Time (min)		
		0 (Basal)	30	60
Serum cortisol (μg/dL)	4.5–21.1	1.5	6.0	6.8
Aldosterone (ng/dL)	3.0–15.9	6.2	10.1	11.8

Synthetic adrenocorticotropic hormone (ACTH) 1–24 (cosyntropin hydroxide 0.25 mg) was administered intravenously in the morning (9 AM)

**Table 3:** Corticotropin-releasing hormone/growth hormone-releasing factor/luteinizing hormone-releasing hormone stimulation test in July 2016 (day 5 after admission)

	Reference range for basal value	Time (min)					
		0 (Basal)	15	30	60	90	120
Serum GH (ng/mL)	0–0.17	0.03	0.41	1.11	1.30	0.92	0.35
Plasma ACTH (pg/mL)	7.2–63.3	15.4	90.1	91.7	75.8	66.5	60.4
Serum cortisol (μg/dL)	4.5–21.1	1.1	2.7	4.5	5.1	5.2	4.9
Serum LH (mIU/mL)	0.8–5.7	0.5	1.0	1.4	1.7	2.0	1.9
Serum FSH (mIU/mL)	2.0–8.3	2.0	2.9	3.1	3.8	3.9	4.0

The following were administered intravenously in the morning (9 AM): 100 μg corticotropin-releasing hormone (CRH), 100 μg growth hormone-releasing factor (GRF), and 100 μg luteinizing hormone-releasing hormone (LHRH)

Our patient had low serum levels of insulin-like growth factor 1 (15 ng/mL; reference range, 48–177) and free testosterone (<0.1 pg/mL; reference range, 4.6–16.9)

*ACTH* arenocorticotropic hormone, *GH* growth hormone, *FSH* follicle-stimulating hormone, *LH* luteinizing hormone

**Table 4:** Growth hormone-releasing peptide-2 stimulation test in July 2016 (day 7 after admission)

	Reference range for basal value	Time (min)				
		0 (Basal)	15	30	45	60
Serum GH (ng/mL)	0–0.17	0.03	0.86	0.86	0.51	0.29
Plasma ACTH (pg/mL)	7.2–63.3	11.2	85.5	68.3	48.1	33.8
Serum cortisol (μg/dL)	4.5–21.1	2.0	4.1	5.8	5.7	5.8

Growth hormone-releasing peptide (GHRP)-2 (100 μg) was administered intravenously in the morning (9 AM)

*ACTH* arenocorticotropic hormone, *GH* growth hormone

**Table 5:** Prolonged arenocorticotropic hormone stimulation test in July 2016 (days 9 to 12 after admission)

	Reference range	Before	After 3 days
Urinary free cortisol excretion (μg/day)	26.0–187.0	7.7	529.5
Basal serum cortisol (μg/dL)	4.5–21.1	0.8	28.6
Basal plasma ACTH (pg/mL)	7.2–63.3	19.7	<1.0

Blood and urine samples were taken with the patient at the supine position each morning (9 AM) on the 2 days before and after 3 days of intramuscular administration of synthetic ACTH 1–24 (cosyntropin zinc hydroxide 1.0 mg/day)

*ACTH* arenocorticotropic hormone

**Table 6:** Thyrotropin-releasing hormone stimulation test in August 2016 (day 21 after admission)

	Reference range for basal value	Time (min)					
		0 (Basal)	15	30	60	90	120
Serum TSH (μIU/mL)	0.5–5.0	1.78	4.16	5.24	5.52	5.24	4.65
Serum prolactin (ng/mL)	3.6–12.8	23.5	49.4	52.2	50.9	44.8	41.7

Thyrotropin-releasing hormone (TRH; 500 μg) was administered intravenously in the morning (9 AM). The test was conducted 21 days after the discontinuation of oral levothyroxine replacement (100 μg/day). Our patient had low serum levels of free triiodothyronine (1.30 pg/mL) and free thyroxine (0.69 ng/dL)

*TSH* Thyro﻿id-stimulating hormone

A brain MRI scan performed in December 2016 showed a 2.5-cm pituitary tumor containing a mixed pattern of solid and liquid components, comparable to that observed 5 months previously. A chest CT scan detected no abnormalities in the lung or heart and no pleural effusion. An echocardiogram showed normal left ventricular wall motion with a LVEF of 71%, moderate mitral valve regurgitation, and no pericardial effusion, and thallium myocardial perfusion scintigraphy with adenosine triphosphate disodium infusion detected no abnormalities.

In March 2017, our patient’s bodyweight, BP, and pulse rate were 69.2 kg, 118/43, and 73 beats per minute, respectively, under dietary salt restriction (6 g/day). Blood chemistry performed following the discontinuation of oral hydrocortisone for 1 day revealed the following; TSH, 0.19 μIU/mL; FT_4_, 1.53 ng/dL; prolactin, 13.8 ng/mL; ACTH, 12.4 pg/mL; cortisol, 0.8 μg/dL; dehydroepiandrosterone sulfate, 34 ng/mL; aldosterone, 19.3 ng/dL; plasma renin activity, 0.3 ng/mL/h; and plasma BNP, 79.3 pg/mL.

His clinical course has been uneventful during replacement therapy with oral hydrocortisone (20 mg/day) and levothyroxine (75 μg/day) for his central AI and hypothyroidism caused by anterior hypopituitarism.

## Discussion

An elderly patient with controlled essential hypertension developed acute anorexia, loss of bodyweight, fatigue, lethargy, and severe periorbital edema with facial swelling in the absence of headache, altered mental status, visual disturbance, and cranial nerve palsy. His periorbital edema resolved within 1 month of treatment with diuretics and levothyroxine replacement for hypothyroidism. He had persistent anorexia, loss of bodyweight, fatigue, and lethargy, and was diagnosed with anterior hypopituitarism, yielding central hypothyroidism and AI, with MRI findings of previous PTA due to hemorrhage in a preexisting pituitary adenoma. He also had laboratory and imaging findings of mild cardiac dysfunction, such as high plasma BNP levels and low LVEF on echocardiogram. Replacement therapy with both levothyroxine and corticosteroids for his hypothyroidism and AI relieved his anorexia, fatigue, lethargy, and cardiac dysfunction. Because hypothyroidism can cause reversible periorbital edema, often accompanied by facial swelling, and cardiac dysfunction [5], his periorbital edema and cardiac dysfunction may have been caused mainly by central hypothyroidism. The present case demonstrates that periorbital edema is an unusual predominant manifestation of PTA.

The pathogenesis of PTA is not fully understood, and in most cases, there is no clear cause. However, known potential precipitants include hypertension, hypotension, diabetes mellitus, major surgery, anticoagulation or clotting disorders, head trauma, and radiation therapy [1, 2]. In the present case, although the patient’s BP appeared to be adequately controlled with antihypertensive medications, his essential hypertension might have been involved in the development of PTA.

The pathophysiology of hypopituitarism due to PTA may include rapid mechanical compression of portal vessels and the hypophyseal stalk, and ischemic necrosis of portions of the anterior lobe. Increases in intrasellar pressure can also cause reduced blood flow through the portal vessels and the hypophyseal stalk, resulting in diminished delivery of hypothalamic hormones to the anterior pituitary [2, 6]. In the present case, the administration of exogenous hypothalamic hormones, including GRF, TRH, LHRH, and CRH, caused incomplete secretion of pituitary GH, TSH, LH, and FSH, and apparently adequate ACTH secretion without sufficient cortisol response. Therefore, our patient’s anterior hypopituitarism was probably due to a combination of impaired pituitary somatotroph, thyrotroph, and gonadotroph, and diminished delivery of endogenous hypothalamic GRF, TRH, LHRH, and CRH to the anterior pituitary, which resulted from both increased intrasellar pressure, and mechanical compression of portal vessels and the hypophyseal stalk. In addition, his mild hyperprolactinemia was mainly due to diminished delivery of hypothalamic dopamine to the anterior pituitary [10].

## Conclusions

Our patient developed acute anterior hypopituitarism in association with PTA, and exhibited severe periorbital edema and mild cardiac dysfunction. The replacement of both corticosteroids and thyroid hormone relieved all of his symptoms of AI, hypothyroidism, periorbital edema, and cardiac dysfunction. Acute central hypothyroidism was probably involved in the development of his periorbital edema and cardiac dysfunction. The present case highlights the need for physicians to consider periorbital edema as an unusual predominant manifestation of PTA.

## Abbreviations

ACTH: Adrenocorticotropic hormone
AI: Adrenal insufficiency
APTT: Activated partial thromboplastin time
BNP: Brain natriuretic peptide
BP: Blood pressure
CRH: Corticotropin-releasing hormone
CT: computed tomography
FT_3_: Free triiodothyronine
FT_4_: Free thyroxine
GH: Growth hormone
GRF: Growth hormone-releasing factor
LHRH: Luteinizing hormone-releasing hormone
LVEF: Left ventricular ejection fraction
MRI: Magnetic resonance imaging
PTA: Pituitary tumor apoplexy
SpO_2_: Oxygen saturation by pulse oximeter
TRH: Thyrotropin-releasing hormone
TSH: Thyroid-stimulating hormone
